# Neonatal corticosteroid therapy affects growth patterns in early infancy

**DOI:** 10.1371/journal.pone.0192162

**Published:** 2018-02-12

**Authors:** Deodata Tijsseling, Maike ter Wolbeek, Jan B. Derks, Willem B. de Vries, Cobi J. Heijnen, Frank van Bel, Eduard J. H. Mulder

**Affiliations:** 1 Division of Woman & Baby, Department of Obstetrics, University Medical Center Utrecht, Utrecht, The Netherlands; 2 Division of Woman & Baby, Laboratory of Neuroimmunology and Developmental Origins of Disease, University Medical Center Utrecht, Utrecht, The Netherlands; 3 Division of Woman & Baby, Department of Neonatology, University Medical Center, Utrecht, Utrecht, The Netherlands; Hopital Robert Debre, FRANCE

## Abstract

**Objective:**

Although postnatal corticosteroid (CS) therapy has well established beneficial effects on pulmonary function, it may also result in growth restriction during treatment. The course of early childhood growth is believed to predict cardiovascular and metabolic diseases in adulthood. Therefore, we determined the effects of postnatal dexamethasone (DEX) or hydrocortisone (HC) treatment on patterns of postnatal growth until approximately four years of age.

**Study design:**

In an observational cohort study of children born prematurely (<32 weeks of gestation), we compared growth patterns for body weight, height, and head circumference from birth to age four years, of children who received DEX (boys: N = 30, girls: N = 14), HC (boys: N = 33, girls: N = 28) to a reference group that had not received postnatal CSs (boys: N = 52, girls: N = 53) using linear mixed-effects modeling.

**Results:**

Growth velocity curves of CS-treated neonates showed a shift to the right, representing a delay in time. They had decreased absolute growth velocities during and shortly after treatment, followed by an increase in growth velocity thereafter. A shift to the right was also seen for the age at which maximal growth velocity of weight/height was reached in boys and girls. Fractional growth rates of weight, height, and head circumference were generally reduced in the CS-treated groups during the first two months of age, with catch-up growth in the following months. In DEX-treated infants these changes were more pronounced than in HC-treated infants.

**Conclusion:**

These data suggest that postnatal growth patterns of preterm born infants are affected by CS-treatment, more by DEX than by HC. Effects were observed mainly on growth velocities. This observation may have impact on health in later life for those individuals treated with CSs in the neonatal period. A definitive conclusion would require a randomized trial of these therapies.

## Introduction

Chronic lung disease (CLD) is a significant problem in preterm infants carrying a high risk of mortality and long-term morbidity [1]. Corticosteroids (CS), especially dexamethasone (DEX) and less frequently hydrocortisone (HC), are used to prevent and reduce CLD. Short-term benefits of neonatal CS-therapy to enhance pulmonary development are well-established with the use of either drug [2–5]. However, follow-up studies of children treated with DEX in the neonatal period have reported on increased rates of cerebral palsy and long-term motor deficits and neuropsychological impairments [6–11]. HC is considered to be safer [12–14], although impairment in neurodevelopment at school age has been described in a small follow-up study [15].

Neonatal DEX has also been associated with impairment of somatic growth, both during treatment (‘early growth retardation’) [16,17] and at follow-up of formerly DEX-treated newborns at ages ranging between 2 and 17 years [10,18–20]. Thus far no significant effect of postnatal HC therapy on somatic growth at ages ranging 2–17 has been found [11,15,21–23]. These follow-up studies on growth in (very) preterm CS-treated infants have generally related (single) measurements of body weight, height, and head circumference at study endpoint to normal growth charts for full-term individuals. However, there is increasing awareness that postnatal growth trajectories estimated from multiple measurements differ considerably between (very) preterm and full-term born infants. Bocca-Tjeertes and others showed the former to have lower median weight and height from birth through the first four years [24]. Further, a single biometric measure does not reflect the timing and dynamics of the growth trajectory along which it was attained across postnatal life. Results from several experimental and clinical studies have indicated that mainly rapid weight gain, but also height in infancy is associated with adverse metabolic and cardiovascular outcomes in later life [14,25–31], but also with improved neurodevelopment in infants [32]. It is presently unclear which impact the combined effects of preterm birth and CS-related early growth retardation may have on subsequent growth; whether CS-treated newborns sometime show catch-up growth presumed to underlie long-term health risks; and whether DEX and HC have differential effects on growth which can be anticipated given some differences in pharmacological characteristics between both drugs [33].

The aim of our study, therefore, was to compare growth patterns for weight, height, and head circumference from birth to age four years, between prematurely born children neonatally treated with DEX or HC and a prematurely born untreated group using growth charts especially constructed for preterm infants. Special interest was paid to measures indicative of accelerated growth and its timing.

## Patients and methods

### Study population

This observational cohort study investigates growth patterns from birth to the age of four years in very preterm born infants (< 32 weeks’ gestation) who had been treated during the neonatal period with CSs to reduce CLD or had not received this therapy. They were admitted between December 1993 and July 1997 to the NICUs of four clinics in the Netherlands: Wilhelmina Children’s Hospital at University Medical Centre Utrecht, Utrecht; Leiden University Medical Centre, Leiden; VU University Medical Center, Amsterdam; and Isala Clinics, Zwolle. The study was approved by the research ethics committee of the University Medical Center Utrecht and written informed consent was obtained from all parents or caregivers. The design of the current study was previously described by Karemaker et al., De Vries et al. and Ter Wolbeek et al., [11,21,34–36]. To reduce CLD, HC therapy in a course starting with 5 mg/kg/day tapering off to 1 mg/kg/day over 22 days was used exclusively in one center (Wilhelmina Children’s Hospital at University Medical Centre Utrecht, Utrecht). A course of dexamethasone (DEX) for the same purpose was used at the three other NICUs, starting with 0.5 mg/kg/day tapering off to 0.1 mg/kg/day over 21 days. In each center, the course was sometimes extended or shortened depending on the infant’s response to therapy. Treatment indication in all instances was the impossibility to wean from the ventilator together with prolonged dependency on extra continuous oxygen based on the initial phase of CLD. Regular meetings were held by neonatologists to discuss treatment strategies to ensure that centers did not deviate from the treatment protocol which was clinically accepted nationwide. The decision to treat was always left at the discretion of the attending neonatologist. HC-treated children did not receive DEX as a rescue therapy, and vice versa. A third study group consisting of very preterm born infants who had not received neonatal CSs comprised the ‘untreated’ group. Information on pregnancy and neonatal characteristics, including parity, smoking during pregnancy, antenatal corticosteroid therapy, singleton or twin pregnancy, gestational age at delivery, birth weight, and infant sex was collected from the obstetric database at each center. Neonatal characteristics were taken from NICU medical records: the occurrence of mortality and morbidity, starting date of neonatal CS treatment, duration of ventilator dependency, feeding, and Apgar scores. Children born small for gestational age (SGA) were defined as children whose birth weight was at least 2SD below the mean for the infant’s GA. Neonatal data for weight, length (crown-heel length) and head circumference was systematically reviewed from patient records kept by each NICU until discharge. Biometric follow-up data was available from visits to the hospital or from records in Preventive Child Health Care centers. During their first 4 years, children in the Netherlands routinely have about 15 well-child check-ups. These include the assessment of weight, height and head circumference (the latter until the large fontanel is closed). All measurements were made by well-trained health professionals using standard procedures. Only children with ≥ 3 serial biometric measurements and with at least one measurement obtained after 12 months of age were included. Exclusion criteria were: birth weight below 2 SDs of the mean (SGA), presence of major congenital anomalies, periventricular leukomalacia or periventricular hemorrhage (≥ grade 3) on neonatal cerebral ultrasound [37]. A total of 210 live born infants was eligible distributed over the three study groups as follows: untreated group (n = 105), HC-treated group (n = 61) and DEX-treated group (n = 44).

### Data analysis

Growth curves for weight, height (both 0–4 years) and head circumference (0–1.5 years) at ages adjusted for each completed week of gestation at birth (between 25 and 40 weeks) and for boys and girls separately have been published for a large Dutch study population [24]. Apart from gestational age at birth and infant sex, these growth charts were unadjusted for other potentially confounding factors. All serially obtained measurements on individuals in the present study were converted to SD scores (SDS) by using the gestation- and sex-adjusted growth reference charts. Graphs of the individual course of raw SDS at 0–4 years were drawn, separately for boys and girls. Individual growth velocity was determined as the increment in weight or size between two successive measurements divided by the intervening time interval. The resulting absolute growth rate, expressed as g/wk or cm/wk, between successive ages was assumed to be that for the mid-point of this time period. Absolute growth rate curves were explored for each subject and maximal (peak) velocity and age at maximal velocity were determined. Secondly, fractional growth rate was calculated for each period as the absolute growth rate divided by starting weight or size, multiplied by 100 (%/wk), considered a measure of catch up growth [38].

### Statistics

Data management and statistical analysis were performed using SPSS for Windows (version 20, IBM/SPSS Inc., Chicago, Ill., USA). Results were summarized with the use of standard descriptive statistics: counts and percentages for categorical variables, and means and standard deviations (SD) or medians and interquartile ranges (IQR) for continuous variables as appropriate. Groups were evaluated for equivalence in patient characteristics and outcome measures using the Chi-square test for categorical measures and one-way ANOVA for continuous variables, followed by post hoc comparisons using the Fisher exact test and Dunnet’s correction for multiple comparisons as appropriate. Variables not-normally distributed were transformed (natural log, square root).

The serial measures (SDS; growth velocities; fractional growth rate (FGR; %week^-1^)) available for each infant were analyzed with linear mixed-effects modeling to produce individual growth trajectories. To study infant growth between birth and 1 year in detail, absolute growth velocities for weight and size were subjected to linear mixed-effects modeling (cubic models) and analyzed by sex and treatment group. Age at birth, birth weight, age at start of CS treatment and singleton/twin pregnancy were included in the models as potential covariates. Mixed-effects models were applied to the serial SDS in two steps, from birth to 12 months and from 12 to 48 months. Data were best fitted by a cubic function of age on step 1 (see S1 Table for model estimates) and by a linear or quadratic function of age on step 2 (model estimates not shown). Mixed-effects models allow for intra-infant correlation of repeated measurements make use of the exact age at measurement, and account for a dissimilar number of measurements on each infant. Such models also allow for individual variation in growth trajectories, as random effects permit variability in intercept, slope, and curvature between subjects. For each biometric variable, we explored linear, quadratic and cubic functions of age (time at birth; t = 0 months). The age terms were included as both fixed and random effects. Neonatal treatment with CSs was included as a main effect and also as an interaction with the age terms. With all tests, significance was assumed at the level of α = 0.05 (two-sided).

## Results

Participant characteristics are summarized in Table 1 and can also be found in the S1 File. Compared to the untreated group, infants in the treated groups were born earlier, weighed less at birth, and were less frequently a twin member (significant for DEX group). However, the group differences in birth weight disappeared after adjustment for gestational age at birth and gender, as evidenced by the birth weight SD scores. In the neonatal period, CS-treated infants had a higher prevalence of Infant Respiratory Distress Syndrome (IRDS) (grades 1–4) and severe IRDS (grades 3–4) and needed more often artificial ventilation and for a longer period of time than untreated infants. The onset of CS-therapy occurred significantly earlier in the HC group than in the DEX group, however this had no contribution to the model. Duration of CS therapy was not significantly different between both groups and duration was therefore not considered a covariable in the analyses of growth.

**Table 1:** Baseline characteristics of the study population.

	Untreated (N = 105)	Hydrocortisone (N = 61)	Dexamethasone (N = 44)	Significance ¶
*Pregnancy characteristics*				
Nulliparous (n; %)	68 (64.8)	41 (67.2)	26 (59.1)	p = .685
Antenatal corticosteroids (n; %)	62 (59.0)	44 (72.1)	26 (59.1)	p = .205
Twins (n; %)	52 (49.5)	21 (34.4)	10 (22.7) ‡	p < .01
*Neonatal characteristics*				
Gestational age at birth (wk); Median [IQR]	29.1 [28.0–30.4]	28.1 [27.1–29.3]‡	27. 4 [26.7–28.8] ‡	p < .0001
Birth weight (g) Median [IQR]	1170 [958–1403]	1040 [938–1155] ‡	1008 [861–1138] ‡	p < .0001
Birth weight SD score #	0.01 ± 0.91	-0.21 ± 0.74	-0.30 ± 0.78	p = .092
Male gender (n; %)	52 (49.5)	33 (54.1)	30 (68.2)	p = .112
5-min Apgar score; Median [IQR]	9 [7–9]	8 [7–9]	8 [7–9]	p = .186
5-min Apgar score < 7 (n; %)	17 (16.2)	12 (19.7)	10 (22.7)	p = .623
Breast feeding (n; %)	53 (50.5)	27 (44.3)	13 (29.5)	p = .064
Ventilator dependency (n; %)	81 (77.1)	59 (96.7) †	44 (100) ‡	p < .0001
Ventilator dependency (days); Median [IQR]	7 [3–14]	15 [8–22] ‡ *	25 [17–35] ‡	p < .0001
Ventilator dependency ≥ 7 days (n; %)	44 (54.3)	53 (89.8) ‡	43 (97.7) ‡	p < .0001
Age at start CS therapy (days); Median [IQR]		8 [5–16] *	18 [14–27]	P < .0001
Duration of CS therapy (days); Median [IQR]		22 [22.0–22.5]	21 [21.0–21.75]	P = .70
IRDS all grades (n; %) IRDS grade 3–4 (n; %)	57 (54.3) 23 (40.4)	50 (82.0) ‡ 33 (66.0) ‡	35 (79.5) ‡ 25 (71.4) ‡	p < .001 p < .005

Data are Mean ± SD, Median [IQR] or number (%). CS therapy: corticosteroid therapy.

# adjusted for gestational age at birth and gender [24].

¶ One-way ANOVA/Kruskal-Wallis test or Chi-square testing for significant differences across the three study groups.

† p<0.05

‡ p<0.01; post hoc test: hydrocortisone/dexamethasone group *vs*. untreated group (adjusted for multiple comparisons).

* p<0.01; post hoc test: hydrocortisone group *vs*. dexamethasone group (adjusted for multiple comparisons).

We analyzed a total of 3245, 2645, and 1397 measurements for weight, height and head circumference (S2 File), respectively, with an average of 15 (range 3–35), 13 (range 3–26) and 8 (range 3–17) measurements per child for these biometric variables, respectively. The final measurement of both weight and height was made at 41 months (range 15–51), and at 13 months (range 5–18) for head circumference. These numbers and ages did not differ statistically across the study groups or between boys and girls (p-values not shown).

**Fig 1:**
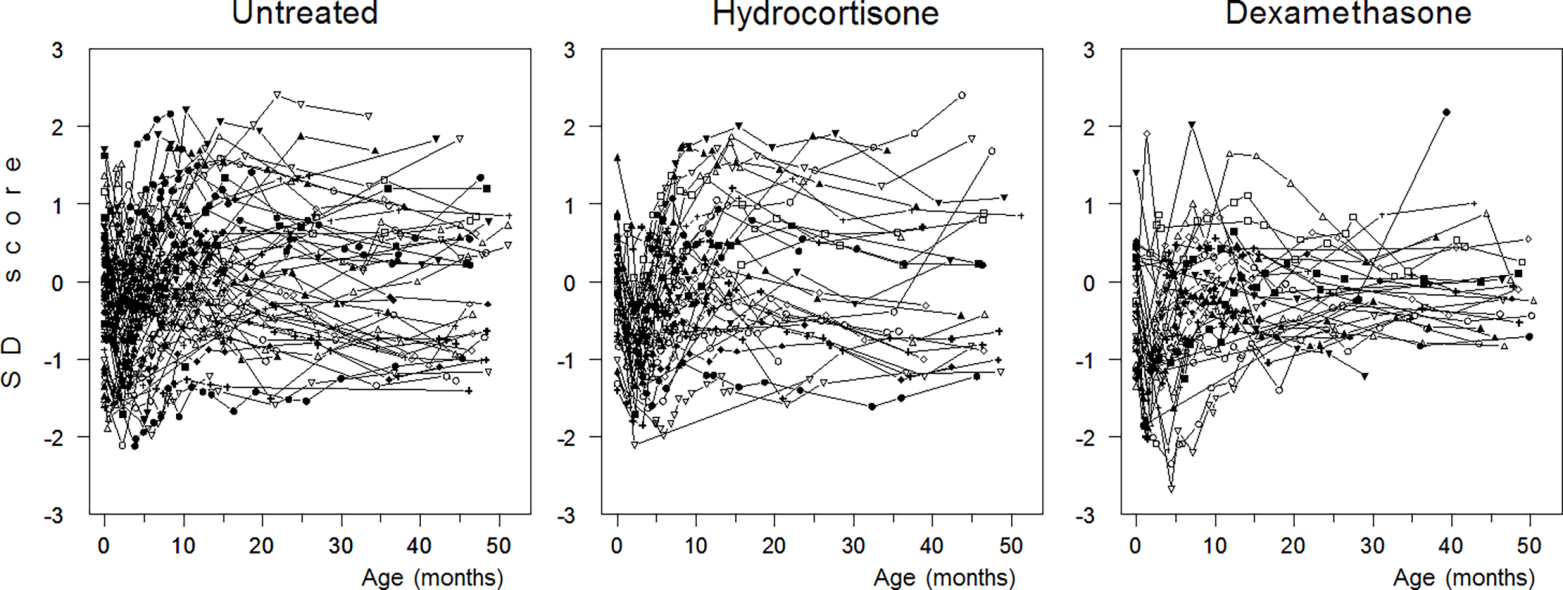
Distribution of measurements and course of body weight SD-scores in individual male children in each study group from birth to 48 months of age.

**Fig 2:**
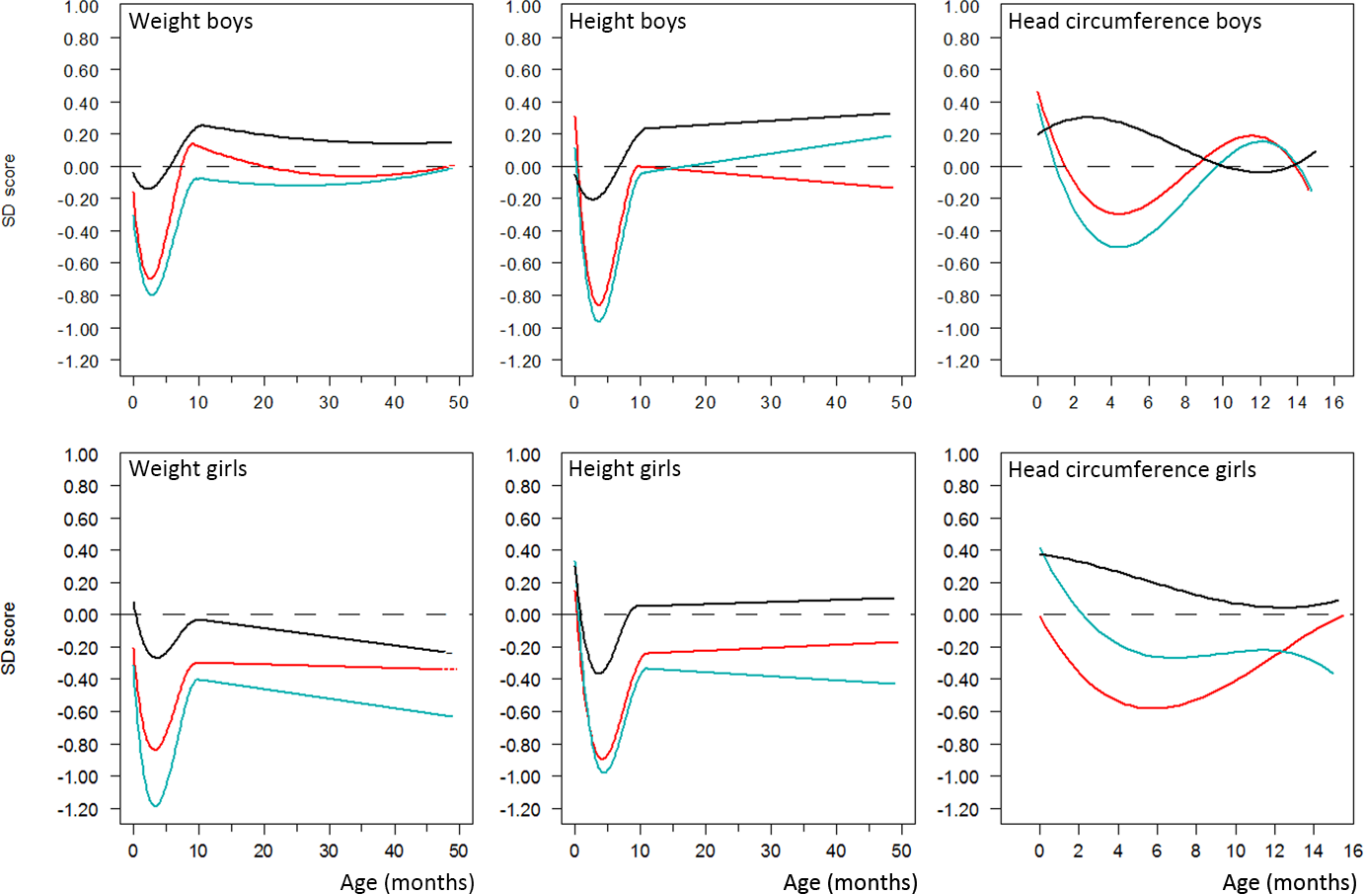
Growth in weight of males (a) and females (b), in height of males (c) and females (d) and in head circumference of males (e) and females (f), ages 0 to 4 in DEX (blue line), HC (red line) and untreated (black line) infants using SD-scores.

Weights and sizes converted into SDS had normal distributions (mean 0, SD 1). Fig 1, as an example, shows the distribution of measurements and course of body weight SDS for individual boys in each study group from birth to 4 years of age. The mean growth trajectories over the first 4 years (weight, height; head circumference till 15 months) are shown in Fig 2 by sex and treatment group. Subsequent to CS therapy, there was a significant fall in SDS for weight and height (about -1 SD) and to a lesser extent for head circumference (about -0.6 SD) at 2–5 months of age. For boys, the growth trajectories of the three groups converged by about one year and showed normal growth thereafter (Fig 2A, 2C and 2E). For girls, the growth trajectories did not fully converge and biometry remained reduced over time in the CS-treated groups, the DEX group in particular (Fig 2B, 2D and 2F). However, results from both steps of analysis showed that the model-predicted SD scores both at birth and at 1 and 4 years of age for both sexes did not differ statistically between the three groups.

**Fig 3:**
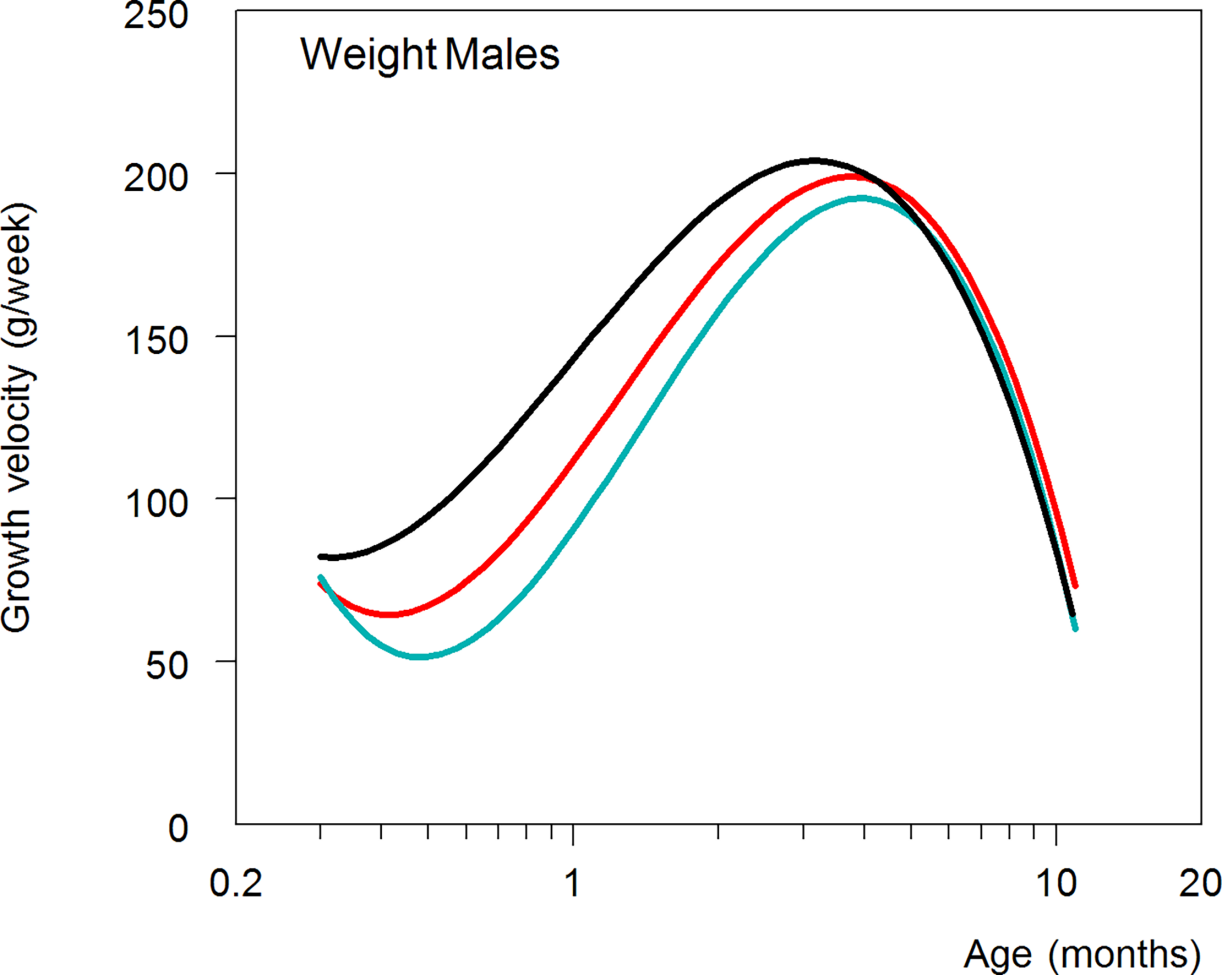
Growth velocity for weight separate for boys in DEX (blue line), HC (red line) and untreated infants (black line). Note that age is on a logarithmic scale and reflects the midpoint of successive measurements, centered at one month of age.

Only birth weight appeared of statistical importance, with lower growth velocities in infants born heavier. Graphic presentation of the six resulting models (estimates not shown) is confined to that for male body weight (Fig 3), as all absolute growth rate curves had similar features. CS-treated infants compared to untreated infants showed significantly lower growth velocities for weight and size at 1 month of age. Thereafter, velocities increased in medicated infants and the shape of their curve of velocity magnitude resembled that seen in untreated infants, although with delay in time (Fig 3). This ‘shift of curves to the right’ in medicated infants was also found for the age at which maximal (peak) growth velocity of weight/size was reached in individual boys and girls, whereas peak velocities, adjusted for birth weight, were generally similar across the study groups, except for weight and head circumference in DEX-treated girls (Table 2).

**Table 2:** Maximal (peak) growth velocity of infant weight and size and age at achievement.

	Males ¶			Females ¶		
	Untreated (N = 52)	Hydrocortisone (N = 33)	Dexamethasone (N = 30)	Untreated (N = 53)	Hydrocortisone (N = 28)	Dexamethasone (N = 14)
*Body weight*						
Age at maximal velocity (months)	3.4 (1.7)	4.2 (1.7)	4.7 (1.8) †	2.9 (1.5)	4.4 (1.6) ‡	5.2 (1.9) ‡ *
Maximal velocity (g/wk)	247 (54)	239 (52)	221 (55)	217 (39)	204 (39)	185 (47) †
*Body height*						
Age at maximal velocity (months)	2.8 (2.1)	3.9 (2.1) †	4.8 (2.1) ‡ *	2.4 (1.4)	3.7 (1.5) ‡	3.9 (1.8) †
Maximal velocity (cm/wk)	1.23 (0.4)	1.15 (0.4)	1.02 (0.4)	1.12 (0.4)	1.13 (0.5)	1.05 (0.5)
*Head circumference*						
Age at maximal velocity (months)	1.6 (1.2)	2.5 (1.1) ‡	3.0 (1.2) ‡	1.7 (0.8)	2.4 (0.9) ‡	3.2 (1.0) ‡ *
Maximal velocity (cm/wk)	0.82 (0.2)	0.75 (0.2)	0.69 (0.2)	0.81 (0.4)	0.80 (0.4)	1.0 (0.7) †

Data are Mean ± SD, adjusted for birth weight.

¶ Multivariable ANOVA testing for significant differences across the three study groups.

† p<0.05

‡ p<0.001; post hoc test: hydrocortisone/dexamethasone group *vs*. untreated group (adjusted for multiple comparisons).

* p<0.01; post hoc test: hydrocortisone group *vs*. dexamethasone group (adjusted for multiple comparisons).

**Fig 4:**
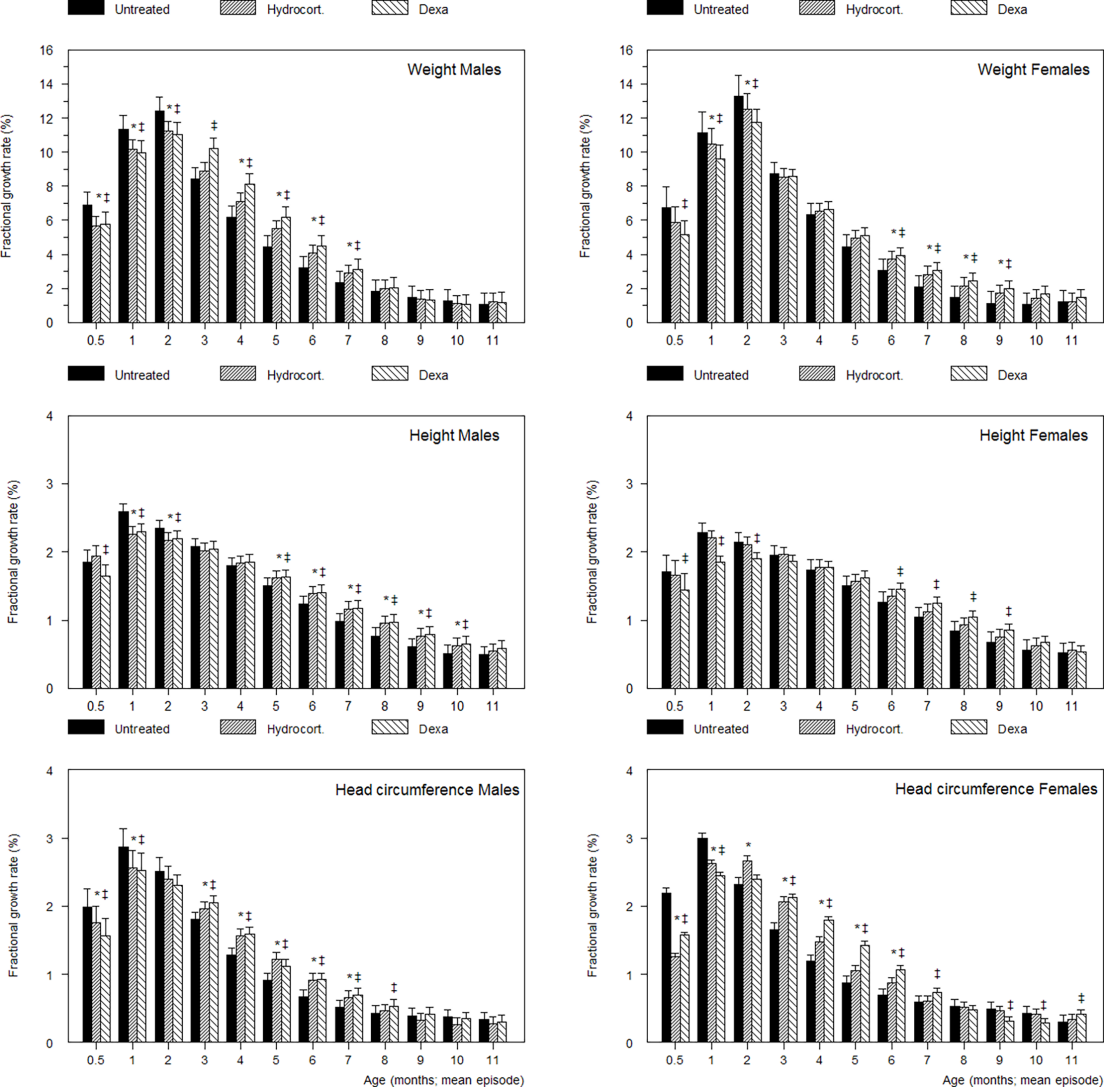
Fractional growth rate (%/week) for weight, height, head circumference in DEX, HC and untreated boys and girls. * P<0.05 HC vs. untreated, ‡ P<0.05 DEX vs untreated, † P<0.05 DEXvs. both, HC and untreated. Analyzed with linear mixed-effects modeling.

Model estimates for fractional growth rate (FGR; %week^-1^) are given in the S2 Table and model-predicted values at 1-month intervals during the first year are presented in Fig 4. Note that age reflects the midpoint of the period for which FGR was determined. In CS-treated infants compared with untreated infants, FGR was generally reduced during the first 2 months, followed by an episode of increased FGR-values for which the timing and duration varied for the three biometric variables and infant sex, but not for the type of CS-treatment. For instance, the onset and end of increased FGR-values for weight was different between boys (3–7 months) and girls (6–9 months) and to some extent also for height (Fig 4).

## Discussion

The current findings suggest that neonatal intervention with DEX and HC affects growth patterns for weight, height, and head circumference of preterm born males and females in their first 48 months, as compared to growth parameters of a preterm born reference group that was not CS-treated. Effects of DEX and HC treatment were observed mainly on growth velocities. Compared to untreated infants, the growth velocity curves of treated neonates showed a shift to the right, representing a delay in time. They presented as decreased absolute growth velocities during and shortly after treatment, followed by an increase in growth velocity. A shift to the right was also seen for the age at which maximal growth velocity of weight/size was reached in boys and girls. FGRs of weight, height, and head circumference were generally reduced in the treated groups during the first two months of age, with catch-up growth in the following months. In DEX-treated infants these changes were more pronounced than in HC-treated infants.

Thus far, it is not clear what the most healthy, optimal growth pattern is and how it can be achieved. It is known that rapid weight gain in infancy, mainly after a period of growth restriction (‘catch-up’ growth) is an important risk factor for subsequent obesity [39,40], coronary heart disease [41], hypertension [25,42] and insulin resistance [43]. Furthermore peak height growth velocity in infancy is reported to be positively associated with blood pressure and waist circumference in adulthood [44]. Therefore, the changes in growth curve shown by DEX and HC treated infants in this study may eventually have consequences for the development and the expression of disease later in life, but, only a long-term follow-up of these patients will provide any answer to this issue.

A mechanism by which accelerated growth makes one increasingly susceptible to cardiovascular and metabolic diseases in adulthood is through alterations in body composition, including a disproportionately high fat mass associated with a lower metabolic rate and insulin resistance [45]. On the other hand, the study of Yang et al. [32] suggests that faster postnatal growth may also have beneficial effects. They found that rapid growth (weight and height) in infancy and early childhood is positively associated with the development of cognition and mental health when tested at the age of 6.5 years, whereas mental illness is among one of the adverse health consequences associated with slower growth [46]. However, the study of Yang et al. was conducted exclusively in full term infants [32].

We also found an effect of CS treatment on head circumference growth. Concurrent with and subsequent to CS therapy there was a fall in SD score for head circumference, with the males converging around the age of 1 year but not the females. Beside this, the age of maximal growth velocity was later in CS-treated infants, while DEX-treated females reached a higher velocity. This effect on head circumference growth could be an indication for poorer neurodevelopment, since a positive association has been reported between head circumference growth on the one hand and neurocognitive development (IQ, school performance) and motor ability in childhood on the other hand for prematurely born and/or very low birth weight infants [12–14]. However, we need to be careful when drawing conclusions based on the DEX female head circumference group because this was a very small group of only 14 children.

Whether the period of reduced FGR with subsequently increased FGR may have an impact on later development has not been studied before. The study of Ghods et al. [14] reported that head circumference catch up growth during the first postnatal year of preterm born infants was positively correlated with neurodevelopmental outcome later in life.

However, all conclusions of the above-mentioned growth studies are made independently of CS-treatment. If DEX and HC do increase the risk of long term health consequences, e.g. by inducing poor growth during the first month and increased growth velocity thereafter, this would be an interesting subject of further study.

Growth impairment during high-dose CS treatment has a multifactorial nature as CSs interfere with growth through multiple pathways [47]. Differences in impact on FGR and maximal velocity between DEX and HC may be caused by the pharmacological and biological characteristics of the two CSs, such as differences in binding preference to the glucocorticoid receptor (DEX) or mineralocorticoid receptor (HC) [48]. Moreover, because DEX and HC differ in potency, the dose of DEX administered in this study is 2.5 times more potent than the HC dose. Therefore, we cannot conclude that DEX has more detrimental effects than HC. Fewer effects on growth in the HC-treated infants may indicate overtreatment in the DEX-treated group. However, the DEX treatment schedule used in this study was comparable to treatment schedules at other NICUs in that period of time [49].

The major strength of this study is primarily its longitudinal approach, involving a minimum of three repeated growth measurements. Former studies reporting on the effects of neonatal CS-therapy on growth compared time points not growth patterns [10,11,18–23]. A second strength is the use of reference growth curves specifically developed for preterm born infants and built up from longitudinal data for weight, height, and head circumference [24]. Previously, reference growth charts for preterm infants were often based on cross-sectional data [50–52].

Limitations of this study include the use of small groups, especially the group of DEX treated females. A lot more children were included in the original NEOCORT database [11,21,34–36]. However, to compare growth patterns we used strict inclusion criteria, e.g. that individuals needed to have at least 3 serial biometric measurements to be included in the analysis. Furthermore, we did not have information about factors that are known to influence neonatal growth, including parental height, feeding problems, infections and other neonatal complications [53–55]. Finally, our study had a retrospective design. A major problem of these studies is to find a comparable untreated group because infants treated with corticosteroids are often born more prematurely, at a lower birth weight, and have more serious (pulmonary) morbidity. Ideally, a randomized controlled trial should be undertaken to investigate the effects of neonatal DEX and HC on growth. However, in view of the effects of DEX courses used in the nineties, this is no longer possible since the dose regimen for the use of DEX has changed. Even so, we are confident that our data are reliable, because measurements were done with standardized equipment and techniques, and by professionals who were trained for measuring children. In conclusion, our findings suggest that growth patterns of preterm born infants were affected by CS-treatment, more by DEX than by HC. The findings may have impact on health in later life for those individuals treated with CSs in the neonatal period.

## Supporting information

S1 Table Statistical results of linear mixed modeling for growth SD scores between 0 and 12 months. Data are presented as estimates (SE) of the models. ‡ p < 0.0001; ¶ p < 0.001; † p < 0.005; * p < 0.05
(DOCX)

S2 Table Statistical results of linear mixed modeling for fractional growth rate between 0 and 12 months. Age centred at 1 month. Data are presented as estimates (SE) of the models. ‡ p < 0.0001; ¶ p < 0.001; † p < 0.005; * p < 0.05
(DOCX)

S1 File Participant characteristics. (SAV)

S2 File Measurements for weight, height and head circumference. (SAV)
