# Supplementary material for: Neonatal corticosteroid therapy affects growth patterns in early infancy
Source: PLoS One. 2018 Feb 12;13(2):e0192162. doi: 10.1371/journal.pone.0192162 (PMC5809117; doi:10.1371/journal.pone.0192162)
Supplement: S1 Table — Data are presented as estimates (SE) of the models. ‡ p < 0.0001; ¶ p < 0.001; † p < 0.005; * p < 0.05 (DOCX) [file pone.0192162.s001.docx]

**S1 Table**

| Variable | Body Weight | | Body Height | | Head Circumference | |
| --- | --- | --- | --- | --- | --- | --- |
|  | Boys | Girls | Boys | Girls | Boys | Girls |
| Intercept | -0.116 (0.108) | 0.116 (0.113) | -0.084 (0.144) | 0.285 (0.142) * | 0.205 (0.127) | 0.375 (0.140) † |
| *Main Effects* |  |  |  |  |  |  |
| Treatment  Hydrocortisone (Group 2)  Dexamethasone (Group 3)  Untreated group (Group 1) | -0.065 (0.173)  -0.219 (0.179)  0 (0) | -0.332 (0.192)  -0.425 (0.241)  0 (0) | 0.431 (0.253)  0.196 (0.347)  0 (0) | 0.019 (0.233)  0.094 (0.363)  0 (0) | 0.244 (0.205)  0.216 (0.223)  0 (0) | -0.398 (0.236)  0.070 (0.301)  0 (0) |
| Time  Age linear  Age squared  Age cubic | -0.152 (0.040) ‡  0.044 (0.0085) ‡  -0.0024 (0.0005) ‡ | -0.237 (0.041) ‡  0.054 (0.0074) ‡  -0.0028 (0.0004) ‡ | -0.216 (0.064) ¶  0.0513 (0.012) ‡  -0.0027 (0.00069) ‡ | -0.499 (0.055) ‡  0.093 (0.012) ‡  -0.0046 (0.0007) ‡ | -0.0047 (0.048).  -0.0049 (0.008).  0.00032 (0.0004) | -0.088 (0.047)  0.0087 (0.0081)  -0.00028 (0.0004) |
| *Interaction Effects* |  |  |  |  |  |  |
| Group 2 x Age linear  Group 3 x Age linear  Group 1 x Age linear | -0.239 (0.065) ‡  -0.219 (0.073) †  0 (0) | -0.184 (0.067) †  -0.161 (0.088)  0 (0) | -0.483 (0.120) ‡  -0.363 (0.171) *  0 (0) | -0.344 (0.089) ‡  -0.352 (0.149) *  0 (0) | -0.422 (0.079) ‡  -0.399 (0.091) ‡  0 (0) | -0.256 (0.079) ¶  -0.413 (0.122) ¶  0 (0) |
| Group 2 x Age squared  Group 3 x Age squared  Group 1 x Age squared | 0.046 (0.014) ¶  0.047 (0.016) †  0 (0) | 0.039 (0.012) ¶  0.026 (0.016)  0 (0) | 0.081 (0.021) ¶  0.056 (0.029)  0 (0) | 0.054 (0.019) †  0.052 (0.029)  0 (0) | 0.073 (0.014) ‡  0.074 (0.016) ‡  0 (0) | 0.041 (0.014) †  0.070 (0.021) ¶  0 (0) |
| Group 2 x Age cubic  Group 3 x Age cubic  Group 1 x Age cubic | -0.0023 (0.0008) †  -0.0025 (0.0096) *  0 (0) | -0.0019 (0.0007) †  -0.0009 (0.0009).  0 (0) | -0.0038 (0.0011) ¶  -0.0023 (0.0015)  0 (0) | -0.0023 (0.0011) *  -0.0021 (0.0016)  0 (0) | -0.0031 (0.0007) ‡  -0.0034 (0.0008) ‡  0 (0) | -0.0016 (0.0006) *  -0.0030 (0.001) †  0 (0) |
