# Supplementary material for: Neonatal corticosteroid therapy affects growth patterns in early infancy
Source: PLoS One. 2018 Feb 12;13(2):e0192162. doi: 10.1371/journal.pone.0192162 (PMC5809117; doi:10.1371/journal.pone.0192162)
Supplement: S2 Table — Age centred at 1 month. Data are presented as estimates (SE) of the models. ‡ p < 0.0001; ¶ p < 0.001; † p < 0.005; * p < 0.05 (DOCX) [file pone.0192162.s002.docx]

**S2 Table**

| Variable | Body Weight | | Body Height | | Head Circumference | |
| --- | --- | --- | --- | --- | --- | --- |
|  | Boys | Girls | Boys | Girls | Boys | Girls |
| Intercept | 22.71 (1.05) ‡ | 22.36 (0.75) ‡ | 3.34 (0.21) ‡ | 2.95 (0.24) ‡ | 5.06 (0.19) ‡ | 4.77 (0.24) ‡ |
| *Main Effects* |  |  |  |  |  |  |
| Treatment  Hydrocortisone (Group 2)  Dexamethasone (Group 3)  Untreated group (Group 1) | -5.01 (1.56) ¶  -2.74 (1.70) n.s.  0 (0) | -2.26 (0.74) †  -2.73 (0.94) †  0 (0) | -0.55 (0.20) †  -0.53 (0.29) *  0 (0) | -0.19 (0.24) n.s.  -0.75 (0.34) *  0 (0) | -1.29 (0.26) ‡  -0.68 (0.30) *  0 (0) | 0.82 (0.40) *  -1.82 (0.49) ‡  0 (0) |
| Time  Age linear  Age squared  Age cubic | -4.95 (0.56) ‡  0.46 (0.096) ‡  -0.0146 (0.0048) ¶ | -4.64 (0.38) ‡  0.37 (0.065) ‡  -0.009 (0.0034) ‡ | -0.164 (0.092) *  -0.029 (0.015) *  0.0023 (0.0007) ¶ | -0.047 (0.109) n.s.  -0.036 (0.017) *  0.0023 (0.0008) † | -1.27 (0.098) ‡  0.131 (0.016) ‡  -0.0046 (0.0008) ‡ | -1.302 (0.134) ‡  0.151 (0.024) ‡  -0.0060 (0.00120) ‡ |
| Birth weight | -0.0025 (0.00038) ‡ | -0.0024 (0.00033) ‡ | -0.00044 (0.00008) ‡ | -0.00053 (0.0001) † | -0.00039 (0.00007) ‡ | -0.00036 (0.00009) ‡ |
| *Interaction Effects* |  |  |  |  |  |  |
| Group 2 x Age linear  Group 3 x Age linear  Group 1 x Age linear | 2.85 (0.92) ¶  2.46 (1.01) *  0 (0) | 0.73 (0.26) †  0.87 (0.35) *  0 (0) | 0.178 (0.06) †  0.164 (0.09) *  0 (0) | 0.055 (0.081) n.s.  0.230 (0.109) *  0 (0) | 0.780 (0.156) ‡  0.437 (0.168) †  0 (0) | -0.167 (0.242) n.s.  1.325 (0.309) ‡  0 (0) |
| Group 2 x Age squared  Group 3 x Age squared  Group 1 x Age squared | -0.445 (0.156) †  -0.443 (0.172) *  0 (0) | -0.051 (0.021) *  -0.058 (0.028).*  0 (0) | -0.012 (0.0047) *  -0.011 (0.0063) n.s.  0 (0) | -0.0037 (0.006) n.s.  -0.0157 (0.0078) *  0 (0) | -0.122 (0.026) ‡  -0.069 (0.027) *  0 (0) | 0.0037 (0.042) n.s.  -0.231 (0.056) ‡  0 (0) |
| Group 2 x Age cubic  Group 3 x Age cubic  Group 1 x Age cubic | 0.0204 (0.0078) *  0.0216 (0.0086) *  0 (0) | n.s.  n.s.  0 (0) | n.s.  n.s.  0 (0) | n.s.  n.s.  0 (0) | 0.0055 (0.0013) ‡  0.0031 (0.0013) *  0 (0) | 0.00041 (0.0022) n.s.  0.0114 (0.0031) ‡  0 (0) |
